# End-stage renal disease secondary to anti-glomerular basement membrane disease in a child with common variable immunodeficiency 

**DOI:** 10.5414/CNCS109510

**Published:** 2019-02-01

**Authors:** Sai Sudha Mannemuddhu, William Clapp, Renee Modica, Melissa E. Elder, Kiran Upadhyay

**Affiliations:** 1 Division of Pediatric Nephrology, Department of Pediatrics,; 2 Division of Anatomic Pathology, Department of Pathology, and; 3 Division of Pediatric Allergy, Immunology, Rheumatology, Department of Pediatrics, University of Florida, Gainesville, FL, USA

**Keywords:** anti-glomerular basement membrane antibody, rapidly-proliferative glomerulonephritis, end-stage renal disease, common variable immunodeficiency

## Abstract

Background: Anti-glomerular basement membrane (GBM) disease is caused by autoantibodies against the α3-chain of type IV collagen in the GBM. Common variable immunodeficiency (CVID) is a primary immunodeficiency manifested by hypogammaglobulinemia, inability to make functional antibody, and recurrent infections. This report extends the phenotype of CVID-associated autoimmune diseases to include anti-GBM disease. Case presentation: A 15-year-old Caucasian female with prior normal renal function presented with nephrotic proteinuria, pedal edema, oliguria, acute kidney injury, and was found to have positive serum anti-GBM antibody. She had been diagnosed with CVID at 3 years of age. Her renal biopsy showed crescentic glomerulonephritis (50%), and immunofluorescence showed linear staining for IgG along the glomerular capillary wall. There was no clinical or imaging evidence of pulmonary hemorrhage. She was treated with pulse IV steroids, cyclophosphamide, rituximab, and several sessions of plasmapheresis. Her serum anti-GBM antibody level decreased from 194 U/mL at presentation to 0 U/mL after therapy. However, she progressed to end-stage renal disease (ESRD) within weeks, despite aggressive therapy, and required chronic renal replacement therapy in the form of dialysis. Her clinical course was also complicated by hypertensive encephalopathy, CMV viremia and meningoencephalitis, status epilepticus, and she passed away a few months later from lower respiratory tract complications. Conclusion: Anti-GBM disease is a rare autoimmune condition that has not been reported in association with a primary immunodeficiency syndrome. ESRD secondary to anti-GBM disease in a patient with CVID is an interesting association and supports the role of immune dysregulation in systemic autoimmune disease.

## Introduction 

Anti-glomerular basement membrane (GBM) disease is a rare autoimmune disorder in which circulating antibodies are directed against an antigen normally present in the GBM, alveolar basement membrane, or both [1]. These anti-GBM antibodies cause rapidly progressive glomerulonephritis (GN), pulmonary hemorrhage, or both. Common variable immunodeficiency (CVID) is the most frequently diagnosed primary immunodeficiency disease in children [2]. We present a case of a 15-year-old female with CVID who presented with anti-GBM disease leading to end-stage renal disease (ESRD). Although other autoimmune conditions and malignancies are known to occur with CVID, here we describe an interesting association of anti-GBM disease with CVID in a child. 

## Case presentation 

A 15-year-old Caucasian female was diagnosed with CVID at 3 years of age during work-up for recurrent infections and chronic lung disease. During the first 2 years of life, she had multiple hospitalizations for wheezing, pneumonia, bronchiectasis, severe pseudomonas ear infections, and failure to thrive. First wheezing episode occurred at 3 months of age and required chronic home bronchodilator nebulization therapy. Initial immunology work-up revealed low CD4 count, poor T-cell function by mitogen studies, B cells within normal range, and near-normal serum immunoglobulin levels (IgG, IgM, IgA). No numerical details were available to us. She was found to have “functional antibody deficiency” due to absence of detectable titers to tetanus, despite routine childhood immunizations, and no protective antibody titers to *Haemophilus influenzae type b* following at least 3 attempts at revaccination. She was maintained on chronic intravenous (IV) or subcutaneous (SQ) immunoglobulin therapy and did well overall until she developed West Nile meningoencephalitis at the age of 12 years, leading to severe residual motor deficits, in the form of quadriparesis requiring a wheelchair, and cognitive changes. Her CVID treatment included 20% SQ immunoglobulin (Cuvitru, Shire Pharmaceuticals, Lexington, MA, USA) every 2 weeks, but there was evidence of nonadherence. Poor adherence was suggested by not picking up the immunoglobulin from the pharmacy and multiple hospitalizations/urgent care visits during that time for flare of wheezing and ear infections. She presented to the ED with progressive fatigue along with rapid weight gain of 4.5 kg in 6 weeks, and decreased urine output with facial and leg swelling. She was found to have hypertensive urgency with manual BP of > 99^th^ percentile for height. Physical examination showed mild generalized anasarca, no hepatosplenomegaly, no lymphadenopathy, no skin rash, and normal chest examination. Labs showed elevated serum creatinine of 486.2 µmol/L (53 – 97 µmol/L) and BUN 21.7 mmol/L (1.8 – 7.1 mmol/L) along with mild hyperkalemia (6 mmol/L, normal 3.5 – 5 mmol/L) and anion gap acidosis. Her prior renal function testing performed 6 weeks prior during an episode of painless gross hematuria (“cola-colored urine”) had shown normal electrolytes with serum creatinine of 17.68 µmol/L. That episode of gross hematuria lasted for ~ 3 weeks and then resolved spontaneously. She did not have fever, loin to groin or abdominal pain, joint pains, skin rash, sore throat, cough, nausea, hematemesis, vomiting, or diarrhea. Blood pressure was not documented. Urinalysis at that time showed 2+ proteinuria, 703 red cells, 36 white cells, negative nitrites and leukocytes. Urine culture was positive for > 100,000 cfu/mL lactobacillus and 10,000 – 50,000 cfu/mL coagulase-negative staphylococcus. She was treated for urinary tract infection with levofloxacin. Serum complements were not checked. 

In the ED, other labs showed white blood cell count of 12.5 × 10^9^/L (4.5 – 11.0 × 10^9^/L, hemoglobin 8.7 g/dL (12 – 15 g/dL), and platelet count of 402 × 10^9^/L (150 – 450 × 10^9^/L). EKG showed tall peaked T waves, and her hyperkalemia was treated with kayexalate, calcium gluconate, insulin/glucose, sodium bicarbonate, and nebulized albuterol. Renal sonogram showed slightly enlarged echogenic kidneys with right kidney of 13.6 cm and left kidney of 12.7 cm with poor corticomedullary differentiation but no nephroureterolithiasis. In the past, she had a history of left-sided nephrolithiasis. Urinalysis showed ≥ 500 protein, 52 red cells, 4 white cells, negative nitrites, and leukocytes with a specific gravity of 1.019 and pH of 6. Serum albumin was 30 g/L (35 – 50 g/L). A spot urine protein (mg/dL) to creatinine (mg/dL) ratio was 29.4. Hypertension was managed with IV nicardipine, which was later switched to oral antihypertensive agents, including losartan, amlodipine, clonidine, labetalol, and doxazosin. Serum IgG level was 1,273 mg/dL (658 – 1,534 mg/dL), IgM 91 mg/dL (48 – 186 mg/dL), and IgA 320 mg/dL (57 – 300 mg/dL). Both C- and P-ANCAs were negative. Antinuclear antigen was slight positive at 1 : 40 in a speckled pattern with negative anti-double-stranded DNA, anti-Smith and anti-RNP antibodies. SSA and SSB antibodies were negative. Serum anti-GBM antibody (IgG) was elevated at 194 AU/mL (normal: 0 – 19 AU/mL) by multiplex bead assay (ARUP laboratory, Salt Lake City, UT, USA). Serum complements were normal. 

**Figure 1.**
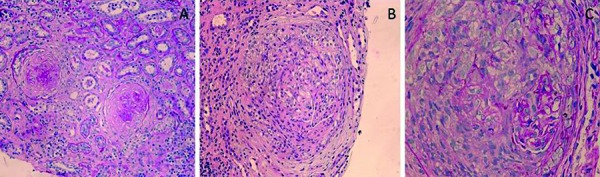
A: Interstitial fibrosis and tubular atrophy and glomeruli showing sclerosis associated with fibrous crescents (PAS, × 200). B: Cellular crescent obliterating glomerular capillaries (H&E, × 200). C: Cellular crescent replacing glomerular tuft. A few residual capillaries are evident (PAS, × 400).

**Figure 2.**
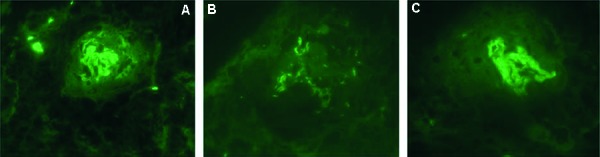
A: Smudgy linear staining for IgG along glomerular capillary walls (× 400). B: Fragments of glomerular capillary wall with linear staining for IgG (× 630). C: Glomerular tuft compressed by fibrous crescent and showing smudgy linear staining for C3 (× 630).

A renal biopsy showed severe glomerular injury characterized by crescentic glomerulonephritis (Figure 1). Out of 11 glomeruli in the light microscopy, 1 glomerulus showed active cellular crescent with progression to fibrous crescents in 3 and fibrocellular crescents in 2 glomeruli along with global glomerulosclerosis (50%). There was patchy, interstitial fibrosis and tubular atrophy, involving at least 40% of the cortical parenchyma. There was acute tubular injury, characterized by patchy tubular dilatation, epithelial attenuation, and luminal casts. Focal moderate intimal thickening of the small arteries was evident without any vasculitis. Immunofluorescence showed 5 globally-sclerotic glomeruli without any intact glomeruli. The glomeruli were negative for IgM, C1q, and albumin. Deeper tissue level immunofluorescence revealed both smudgy and linear staining for IgG, κ-light chain, and λ-light chain in glomerular capillary walls compressed by crescents (Figure 2). Electron microscopy revealed no electron-dense deposits in the available 2 glomeruli. CT of the chest did not show any alveolar hemorrhage, and she never had hemoptysis. Pulmonary function test showed evidence of severe intrinsic restrictive ventilatory dysfunction, most likely related to her neuromuscular illness, unchanged from her pre-illness tests. Diffusion capacity showed anemia and decreased alveolar volume with no evidence of pulmonary hemorrhage or pulmonary fibrosis. 

She was treated with IV methylprednisolone 10 mg/kg/dose for 3 days, 2 doses of cyclophosphamide 1 week apart (10 mg/kg followed by 600 mg/m^2^), rituximab 750 mg/m^2^ q2 weeks × 2 doses and 10 sessions of plasmapheresis every other day. Her anti-GBM antibody titer decreased from 194 to 3 U/mL after the above therapies. She was continued on 60 mg prednisone daily along with azathioprine, following which her anti-GBM level decreased to 0 U/mL (negative). Her prednisone dose was then tapered to 5 mg every other day. She remained without hemoptysis, cough, or respiratory difficulty. However, despite aggressive therapies, she became anuric and progressed to ESRD. Renal replacement therapy in the form of hemodialysis was required early during the course of her illness and was subsequently maintained on nightly chronic peritoneal dialysis. She was discharged home on prednisone, azathioprine, pentamidine IV every 3 – 4 weeks for pneumocystis jirovecii pneumonia prophylaxis, and previously prescribed SQIG (she had been receiving IVIG while an inpatient). Her illness was further complicated by an episode of hypertensive encephalopathy and CMV meningoencephalitis along with CMV viremia requiring IV ganciclovir and then chronic oral valganciclovir therapy. Azathioprine was discontinued as a result. After few months, she developed otitis media, lower respiratory tract infection, candida urinary tract infection, micrococcus septicemia, and status epilepticus, all of which were treated appropriately. A week after her latest hospitalization, her mother informed the office by telephone about her worsening respiratory secretions, despite using vest and cough assist, and postponed the medical care citing extreme weakness of the patient. The following day, she passed away at home, most likely secondary to lower respiratory tract infection. 

## Discussion 

Anti-GBM disease is a rare, small vessel vasculitis condition that affects the capillary beds of the kidneys and presents as rapidly progressive GN. It is a prototypical autoimmune disease, caused by directly pathogenic autoantibodies (mainly of IgG subtype) targeting the noncollagenous-1 (NC1) domains of the α-3 and α-5 chains of type IV collagen, well characterized antigens expressed in the GBM [1]. Anti-GBM disease may occur in isolation or as a component of pulmonary-renal syndrome. In the latter, anti-GBM autoantibodies also cross-react with the alveolar basement membrane and cause similar damage in lungs, presenting as diffuse alveolar hemorrhage and hemoptysis [3]. The terminology “Goodpasture’s disease” is commonly used for the clinical condition characterized by pulmonary hemorrhage and crescentic GN associated with linear deposition of antibodies along the GBM [3]. The pulmonary-renal syndrome could be idiopathic or may occur in association with various forms of primary systemic vasculitis, cryoglobulinemia, and systemic lupus erythematosus. It is important to remember that pulmonary manifestations may be absent in anti-GBM disease [4] as in the patient described in this case report. 

CVID is a primary immunodeficiency condition in which the B lymphocytes fail to differentiate into functional plasma cells that produce the various immunoglobulin isotypes in response to antigen [2]. To define CVID, ICON (International Consensus Document) CVID criteria requires low serum IgG in combination with IgA and/or IgM, along with impaired vaccine response and exclusion of other causes of hypogammaglobulinemia [5]. This heterogeneous disorder also causes T-lymphocyte abnormalities including a reduced T-cell count, decreased lymphocyte proliferation in response to mitogens and antigens, and defective T-cell signaling. These phenotypic and functional T-cell defects may present with a severe phenotype including gastrointestinal tract disease, splenomegaly, granuloma, and lymphoma [6]. Although the onset of symptoms is usually in the third and fourth decades of life, ~ 25% of all CVID patients present in early childhood or adolescence, often from single gene defects, with an earlier peak of diagnosis at ~ 8 years of age [7, 8]. Monogenic forms of CVID, although accounting for only 2 – 10% of patients with CVID, is more likely in early-onset disease (infancy or early childhood), with a positive family history or consanguinity [9]. The patient described in this report did not have a positive family history, and there was no consanguinity. CVID affects males and females equally. It is the most common primary immunodeficiency diagnosed in North America and Europe [2], with an estimated incidence of 1 : 25,000 to 1 : 66,000 in some populations [7]. One study looked at the incidence of CVID in a pediatric tertiary-care center and found that it occurs in 9% of total primary immunodeficiency cases and comprises 20% of antibody deficiencies [5]. Manifestations of CVID in children include chronic respiratory tract infections (88%), rhinosinusitis (78%), otitis media (78%), intestinal tract infections (34%), meningitis, sepsis, pyelonephritis, etc. [7]. 

Renal involvement in CVID includes renal granulomas and IgM glomerulonephritis [11], membranous nephropathy [12], often with resolution after initiating IVIG [13], ESRD of unknown etiology prior to diagnosis of CVID [6], development of post-renal transplant CVID [14], and membranoproliferative GN with normal renal function [11]. Rapidly progressive GN in the setting of CVID resulting in ESRD is uncommon. It is interesting to note that our patient’s anti-GBM antibodies did not cross-react to the alveolar basement membranes. 

Dysregulation of immune processes can lead to autoimmune disorders in primary immunodeficiency [15]. Approximately 1/3 may present with autoimmune diseases, including autoimmune cytopenias, sarcoidosis, autoimmune lung diseases, autoimmune kidney diseases, insulin-dependent diabetes, psoriasis, vitiligo, pernicious anemia, systemic lupus erythematosus, rheumatoid arthritis, juvenile idiopathic arthritis, gluten sensitivity, etc. [10, 16]. Various mechanisms have been proposed in the development of autoimmunity in CVID. These include B-cell receptor editing checkpoint and B-cell maturation defects leading to residual autoreactive B-cell clones, loss of inhibitory signaling, reduced T-regulatory-cell function, and impaired T-helper-cell activity [17]. Recurrent infections in CVID could also contribute to systemic autoimmunity via mechanisms of epitope spreading [18], superantigen activation [19], and bystander activation [20]. We speculate that the recurrent infections in our patient, including pulmonary infections, West Nile meningoencephalitis, and urinary tract infections, could have at least partially contributed to the development of autoantibodies against the type IV collagen of the GBM. 

Treatment of the autoimmune condition, e.g., anti-GBM disease, usually involves immunosuppressive therapy to suppress the causative inappropriate immune responses. However, using aggressive immunosuppression in a patient who already has significant immunodeficiency, e.g., CVID, is complicated by the need to balance effective treatment and development of unwanted infections and other serious side effects. Although SQIG should have decreased her infection risk to some degree, our patient developed infectious complications and progressed to ESRD despite treatment. Although IG is known to result in increased CD4 T-cell numbers, this effect may not be seen in all T-cell subsets. Treg and natural killer T-cells may not be restored with IG infusion; loss of these immune cells may explain why some CVID patients still experience infectious and autoimmune complications [21]. Furthermore, nonadherence to the required IG therapy significantly contributes to these complications in many patients, resulting in adverse life events. Timely initiation of prophylactic therapies and prompt management of infections along with adherence to the IG is very important. Hematopoietic stem cell transplantation may emerge as a potential treatment for CVID [22]. 

## Conclusion 

Autoimmune glomerulonephritis, like anti-GBM disease, may occur in patients with primary immunodeficiency. The treatment required to control the autoimmune process in these patients may further increase the risks of infection or other complications. However, untreated autoimmune disorders like anti-GBM disease are life-threatening with serious morbidities even in patients with immune dysfunction. A multidisciplinary approach is a key in management. 

## Funding 

The authors received no funding for this project. 

## Conflict of interest 

The authors have no conflicts of interest to disclose.
